# School-based intervention to prevent overweight and disordered eating in secondary school Malaysian adolescents: a study protocol

**DOI:** 10.1186/s12889-016-3773-7

**Published:** 2016-10-20

**Authors:** Sharifah Intan Zainun Sharif Ishak, Yit Siew Chin, Mohd. Nasir Mohd. Taib, Zalilah Mohd. Shariff

**Affiliations:** 1 Department of Nutrition and Dietetics, Faculty of Medicine and Health Sciences, Universiti Putra Malaysia, 43400 UPM Serdang, Selangor Malaysia; 2 Research Centre of Excellence, Nutrition and Non-communicable Diseases, Faculty of Medicine and Health Sciences, Universiti Putra Malaysia, 43400 UPM Serdang, Selangor Malaysia

**Keywords:** Adolescent, Disordered eating, Eating disorder, Overweight, Obesity, Malaysia, Intervention, Body image, Physical activity

## Abstract

**Background:**

Obesity, eating disorders and unhealthy weight-loss practices have been associated with diminished growth in adolescents worldwide. Interventions that address relevant behavioural dimensions have been lacking in Malaysia. This paper describes the protocol of an integrated health education intervention namely ‘Eat Right, Be Positive About Your Body and Live Actively’ (EPaL), a primary prevention which aimed to promote healthy lifestyle in preventing overweight and disordered eating among secondary school adolescents aged 13–14 years old.

**Methods/Design:**

Following quasi-experimental design, the intervention is conducted in two secondary schools located in the district of Hulu Langat, Selangor, Malaysia. Adolescents aged 13–14 years will be included in the study. A peer-education strategy is adopted to convey knowledge and teach skills relevant to achieving a healthy lifestyle. The intervention mainly promoted: healthy eating, positive body image and active lifestyle. The following parameters will be assessed: body weight, disordered eating status, stages of change (for healthy diet, breakfast, food portion size, screen viewing and physical activity), body image, health-related quality of life, self-esteem, eating and physical activity behaviours; and knowledge, attitude and practice towards a healthy lifestyle. Assessment will be conducted at three time points: baseline, post-intervention and 3-month follow-up.

**Discussion:**

It is hypothesized that EPaL intervention will contribute in preventing overweight and disordered eating by giving the positive effects on body weight status, healthy lifestyle behaviour, as well as health-related quality of life of peer educators and participants. It may serve as a model for similar future interventions designed for the Malaysian community, specifically adolescents.

**Trial registration:**

UMIN Clinical Trial Registration UMIN000024349 (Date of registration: 11th. October 2016, retrospectively registered).

## Background

Globalisation, urbanisation and economic development in the past century have had a negative impact on eating habits and exercise in both developed and developing nations. This impact is evident in modern adolescents’ eating and physical activity regimens. In Malaysia, adolescents, individuals aged 10–19 years, make up a population of 5.5 million i.e., 18.9 % of the entire Malaysian population [1].

At the age of adolescence, major physical, psychological and social changes occur. For instance, this stage of development is the second chance of catch-up growth before entering adulthood. Inadequate intake of carbohydrates, proteins, fat, vitamins and/or minerals has been shown to have severe consequences on growth during this stage, and is associated with delayed onset of puberty [2]. This is because nutritive demands of adolescence are only second to infancy. Excessive food intake, however, has also been shown to have detrimental effects on adolescents’ health, such as increasing their susceptibility to diseases and precipitating diabetes, heart disease and hypertension [3].

Overweight and obesity are major public health issues in both developed and developing countries. Between 1980 and 2013, the prevalence of overweight and obesity in developed countries remarkably increased from 16.9 and 16.2 % to 23.8 and 22.6 % in male and female adolescents respectively; whereas in developing countries, a whopping 9.8 % increase in the number of overweight and obese adolescents was observed around the same period of time [4].

In Malaysia, high percentages of overweight and obese adolescents, as well as adolescents suffering from disordered eating have been reported. According to the National Health and Morbidity Survey (NHMS), the prevalence of obesity in Malaysian adolescents increased dramatically between 2011 and 2015 from as low as 6.1 % [5] to 11.9 % [6]. Alarmingly, estimates from a separate nationwide study showed that as of 2015, 14.2 % of Malaysian adolescents aged 12–19 were overweight, while 10.1 % were rather obese [7].

Disordered eating refers to unhealthy eating and weight related behaviours and attitudes that are of medical and/or psychological concern but cannot be labelled as eating disorders [8]. In Malaysia, disordered eating is rampant. Approximately 22.3 % of female adolescents in the state of Kelantan [9] and 27.8 % of adolescents in the state of Pahang were found to be suffering from disordered eating [10]. Overweight and obese adolescents were shown to be significantly more prone to disordered eating behaviours than non-overweight, non-obese adolescents of similar age groups [7]. Such behaviours are associated with higher risk of non-communicable and chronic diseases in adulthood. Hence, immediate action to prevent overweight and disordered eating in Malaysia seems to be highly warranted. Obesity and disordered eating share a number of risk factors, such as unhealthy eating attitudes, body dissatisfaction and weight-related teasing among adolescents [11]. Hence, tackling obesity and disordered eating may be achieved using the same approach.

Poor body image can result in negative effects on a child’s or an adolescent’s weight. The physiological changes that happen to the body during adolescence contribute to negative body image [12, 13] and are associated with a multitude of weight-related issues, including obesity, disordered eating, binge eating and extreme weight-control practices [14]. Hence, to be effective, interventions aiming to decrease the incidence of obesity and disordered eating among adolescents should promote positive body image, eating habits and physical activity [15].

This study sought to effect positive changes in the prevalence of overweight and disordered eating in adolescents aged 13–14 years in Form 1 and 2 of secondary school. Most Malaysian adolescents experience puberty and an increase in body fat around this age, which is coincidently the age at which students frequently build new friendships and establish new group norms. This often contributes to body dissatisfaction and unhealthy dietary choices, such as extreme dieting and binging [16, 17], leading to poor overall health status [16]. School students aged 12–15 years are an ideal group to study the effects of universal prevention approaches as the majority of the social, psychological and environmental risk factors that can trigger eating disorders and lead to overweight are most prominent [18]. Therefore, the present peer-group intervention was designed to drive students during this stage of early adolescence to develop attitudes and behaviours that would rather nurture healthy lifestyles.

Achieving nutritional goals, promoting physical activity and positive body image have become the pillars of both obesity and eating disorder prevention programmes. Integrating obesity prevention approaches into behavioural interventions designed to tackle eating disorders has been shown to be more advantageous than separate programmes as it lacks mixed messages and represents a strategy that is more time- and cost-effective [19]. Several integrated interventions implemented around the world have been shown to be successful in the past, such as MABIC [20] and New Moves [21]. New Moves utilised integrated concepts and teaching strategies adopted from both obesity and eating disorder prevention programmes. It promoted physical activity and improved eating patterns in female adolescents by altering negative body image conventions and effecting positive behavioural changes. Another integrated intervention, Healthy Buddies, achieved similar goals in elementary school students, but rather by using a peer-led approach [22].

Healthy Lifestyle in Children (HELIC) [23], Healthy Kids Programme [24], Body Image Education Package [25] and the Malaysian Childhood Obesity Treatment Trial (MASCOT) [26] are a few of many school-based interventions that had been implemented in Malaysia. However, most of these interventions have rather been stand-alone programmes in the sense that they focused either on the prevention of obesity or the prevention of disordered eating. As far as our knowledge goes, Malaysia has not seen a comprehensive intervention programme that promotes all three components of a healthy life: healthy eating, active lifestyle and positive body image.

‘Eat Right, Be Positive About Your Body and Live Actively’ (EPaL) is an integrated health education intervention, which developed to prevent overweight and disordered eating among Malaysian adolescents. This intervention was formulated with the understanding that obesity, disordered eating, and extreme weight loss practices were strongly correlated and shared the same risk factors. Hence, it delivered a coherent message while tackling a broad spectrum of eating- and weight-related health issues. Unlike Healthy Buddies, EPaL did not use a one-to-one approach [22], but rather involved training a group of adolescents to become peer educators who then led others to fully implement the intervention. It differed from MABIC as it stipulated that the trainees be adolescents rather than educators or health care providers, such as tutors, clinical psychologists and nurses [20].

This paper describes the protocol for an integrated health education intervention namely ‘Eat Right, Be Positive About Your Body and Live Actively’ (EPaL), a primary prevention which aimed to promote healthy lifestyle, which including healthy eating, positive body image and active lifestyle, in preventing overweight and disordered eating among first and second year secondary school adolescents. In the present work, adolescents were used to ascertain whether or not EPaL could serve as an effective tool to promote health in Malaysian adolescents and reduce the incidence of overweight and disordered eating. The intervention was developed by taking consideration on risk factors and cultural background of Malaysian population, which might be different from other countries. The risk factors of interest were determined beforehand by carrying out a wide survey amongst secondary school adolescents throughout Malaysia.

## Methods/Design

### Study design

**Fig. 1:**
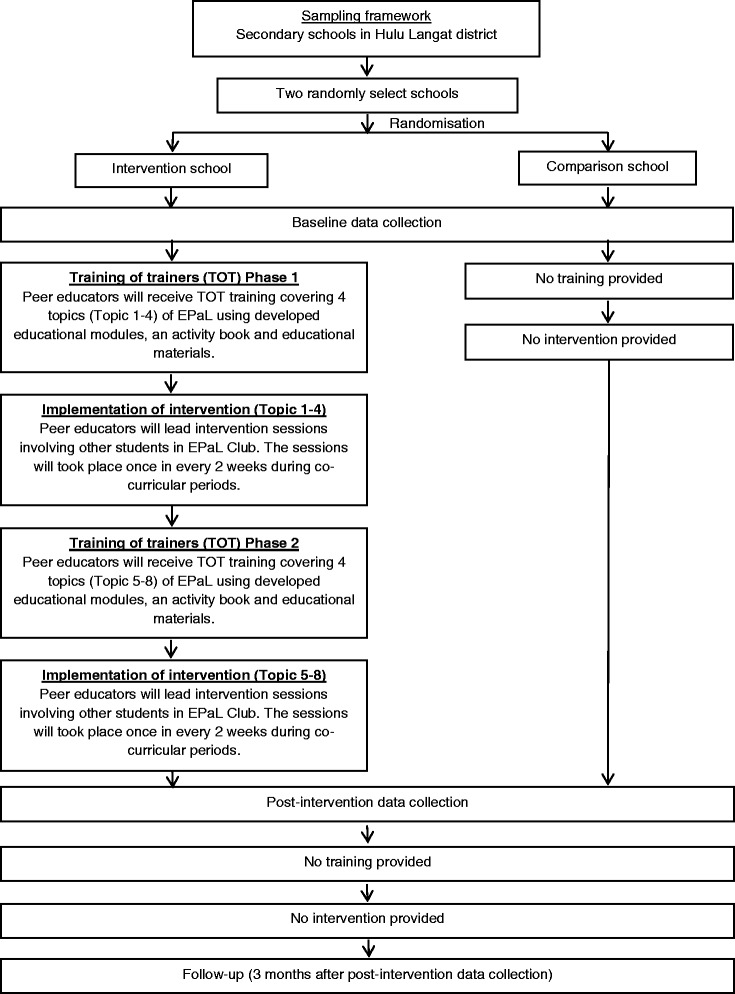
EPaL study design

EPaL is a school-based intervention programme targeting adolescents in the early years of secondary school. The present study adopted a quasi-experimental design, in which adolescents from one school were included in the intervention group, while those from another school served as the comparison group and were not subjected to the intervention until the end of the period of experimentation. Adolescents in the comparison group attended a standard physical and health education class at least once a week. As the study was concluded, they were allowed to receive EPaL modules and educational materials. A flowchart of the intervention is shown in Fig. 1.

### Ethical approval

The study was approved by the Medical Research Ethics Committee of the Faculty of Medicine and Health Sciences at Universiti Putra Malaysia, Selangor, Malaysia. Permission for field data collection in secondary schools was attained from the Malaysian Ministry of Education, as well as the State Department of Education of Selangor. In all cases, consent was obtained from the board of the school, the principal, the parents and the adolescents prior to data collection.

### Setting, recruitment and participants

Two schools were randomly selected in the district of Hulu Langat, the state of Selangor, Malaysia. One school will randomly chose to receive the intervention, while adolescents in the other served as the comparison group. Selection of the schools will be performed by means of a draw from a list of schools in Selangor, obtained from the website of the state’s Department of Education. Only schools that met the inclusion criteria (coeducational, multiracial, non-residential and non-religious) were included in the draw. Adolescents who were Form 1 or 2 students and 13–14 years old; and whose parents consented to the intervention met the criteria for inclusion in the study.

A seminar will be held for the parents and teachers of Form 1 and 2 students in which EPaL will be introduced. Parents and teachers were asked to promote the programme to the students. Recruited students were presented with the research’s information sheet and consent letters to be read and filled by them and their parents. Written consent letters were collected from participating adolescents and their parents prior to the intervention. Thereafter, the students were subjected to the EPaL intervention programme over the course of 16 weeks in their school. They were assessed in three points of assessments, which are prior to the intervention (baseline), after the final session of the programme (post-intervention) and 3 months thereafter (follow-up).

### Sample size calculation

The sample size calculation was based on the differences between groups (overweight and obese; and non-overweight and non-obese) in the proportion of having disordered eating. The value was taken from the outcome of needs assessment study, which was conducted involving 9677 adolescents aged 12–19 years throughout Malaysia. Based on the study, the proportion of overweight and obese adolescents who were having disordered eating was 40.3 %, whereas the proportion of non-overweight and non-obese adolescents who were having disordered eating was 27.9 %. Assuming a power of 80 % and a level of significance of 5 %, we needed 234 participants for each intervention and comparison groups, respectively. The effect size for proportions was estimated by the differences in the arcsine transformations of the respective proportions. The medium effect size of 25.4 % was obtained.

### Description of EPaL intervention

EPaL promoted three main components of healthy lifestyles: (1) healthy eating, (2) positive body image and (3) active lifestyle. The intervention sought to prevent overweight and disordered eating among secondary school adolescents. The rationale of EPaL is that adolescents with positive body image and right response to body signals are more likely to adhere to a healthy lifestyle, eat healthy and stay physically active; and when balance is achieved between energy gained from food and energy lost during physical activity, optimal body weight status ensues.

EPaL adopts the principles of Social Cognitive Theory (SCT) [27], which emphasises the importance of social and environmental factors in determining the psychosocial and behavioural risk factors of both obesity and disordered eating. Hence, EPaL was designed to address socio-environmental factors, such as peer support, presence of a role model, opportunities for practicing healthy eating in/outside the house and opportunities for practice active lifestyle indoors/outdoors; personal factors, such as self-esteem, body image, body weight status/ BMI and self-efficacy of healthy eating and physical activity; and behavioural factors, including self-control/self-regulation, skills to practice healthy eating, physical activity and positive body image, eating behaviour, physical activity behaviour and binge eating behaviour.

EPaL provides students with cognitive and behavioural skills to effect change in targeted behaviours. It aims to alter disordered eating behaviour, promote physical activity, prevent sedentary lifestyle and enhance eating behaviours by encouraging eating in all meal time, consumption of fruits and vegetables and lowering sweetened beverages intake.

According to SCT principles, peers acting as educators should accept increasingly important roles towards developing adolescents’ self-awareness [27]. EPaL intervention are based on the observational learning concepts of SCT and it was designed to engage adolescent educating peers in promoting health by means of cooperation and information sharing. Adolescents tend to accept new information and change their behaviours when observing and learning from their peers [28].

In the present intervention, peer education was used as a strategy to convey health-based knowledge and skills to adolescents. ‘Peer-educators’ referred to students delivering educational material to other students of nearly the same age [29]. For instance, in this study, Form 2 students (14 years old) will deliver the programme to Form 2 and Form 1 students (13 years old). The peer educators will be chosen by the teachers based on academic performance and willingness to convey the intervention message to other students.

All intervention activities will be carried out in a club called ‘EPaL club’, which is established by the research team with the due permission of the school. The purpose of the club is to generate a feeling of belonging among the participants, as well as to allow teachers and the research team to easily monitor the implementation of the intervention.

Prior to the development of EPaL intervention, needs assessments were conducted as follows:

1. Twelve focus groups (*n* = 72) with adolescents aged 13–14 years. The groups engaged in discussions designed to assess the perceptions of adolescents in respect of healthy eating habits, body shape/size and physical activity. The discussions explored strategies that adolescents follow to stay health and the obstacles that prevent adolescents from eating healthy and being active.
2. A nationwide survey was conducted involving 9677 adolescents aged 12–19 years. It aimed to determine the prevalence and factors of underweight, overweight, obesity and disordered eating among Malaysian adolescents.

The EPaL intervention consisted of eight topics which will be delivered by peer educators over the course of 16 weeks, with a two-week gap between every two topics. Each session will take about 60–90 min and the sessions will be held during co-curriculum periods at the school hall in EPaL club. Topics’ titles, key messages and learning outcomes are shown in Table 1.

**Table 1:** Objective, key message and learning outcome of EPaL topics

Topics and aims	Key message	Content
Topic 1 *Know Your Body Image and Body Weight Status* • To promote positive body image. • To promote healthy body weight status.	Achieve positive body image and healthy body weight status	• Presentation: - 5 simple steps to determining body weight status: 1. Measure weight. 2. Measure height. 3. Calculate age in years and months. 4. Calculate body mass index using the given formula. 5. Mark the value of BMI and age on the BMI-for-age chart. - Things to do after determining your own body weight status. - Body image discussion. ▪ Effects of negative body image. ▪ Definition and effects of positive body image. • Activities: - Measure height and weight; calculate age and BMI; and determine your own body weight status. - Compare your own body size perception with actual body weight status and learn about your own body image.
Topic 2 *Achieve Healthy Body Weight* • To identify the concept of energy balance. • To describe ways to eat regularly at specified times during the day.	Understand the needs of your own body and achieve energy balance by eating nutritious foods regularly at specified times during the day.	• Presentation: - Energy balance model. ▪ Ways to achieve energy balance in daily life. - Recommend walking for 10000 steps a day. ▪ Advantages of achieving 10000 steps a day. ▪ Type and duration of exercises comparable to walking 10000 steps. - Introduce 5 main meals: breakfast, morning snack, lunch, afternoon snack and dinner. ▪ Recommended time to consume main meals. ▪ Advantages of eating at specified time points. • Activities: - Introduce the pedometer and discuss how to use it. - Perform physical activity and read the number of steps counted by the pedometer. - Participants share when they usually consume the main meals and peer educators share the recommended time points for main meals during the day.
Topic 3 *Follow the Malaysian Food Pyramid* • To eat in a way that conforms to the concept of SPS (S: seimbang/ balance, P: pelbagai/ variety, S: sederhana/ moderation) and the Malaysian Food Pyramid. • To plan daily menus according to the suggested serving size.	Have balance in food, a variety and eat in moderation to stay healthy.	• Presentation: - Malaysian Food Pyramid: food groups in each level and recommended serving size for each food group. • Activities: - Station game with each station referring to a food group. Interactive activities and hands-on skills related to the respective food group in every station. - Menu planning for one day.
Topic 4 *Active according to Physical Activity Pyramid* • To reducing screen-viewing time. • To increase daily physical activity following the Physical Activity Pyramid.	Reduce screen time and increase physical activity to be healthy and brilliant adolescents.	• Presentation: - Physical Activity Pyramid: physical activities of every level and recommended frequencies. - Definition and examples of sedentary activities. - Screen time to be limited to 2 h daily. • Activities: - Match the physical activity with the frequency in the respective level using an empty Physical Activity Pyramid. - Modify a daily activity for a more active lifestyle by cutting screen time and using the extra time to be active. - Groups of participants race to perform physical activities shown in the Physical Activity Pyramid upon stepping on the pyramid levels drawn on the floor. - Wear pedometer during activities and check the steps count after finishing.
Topic 5 *Choose Your Food Wisely* • To choose healthier foods and beverages by comparing product labels. • To order healthier food and beverages when eating out.	Choose healthier foods based on nutrition labels and cooking methods. Healthy food is not only delicious but also good for your health!	• Presentation: - Food labelling. ▪ Information on food labels. ▪ Steps to read a food label. ▪ Steps to read the nutrition information panel. ▪ Ways to compare and purchase foods and beverages based on the information on the labels. - Tips for healthy eating when eating out. • Activities: - Comparing foods and beverages by consulting the product label. Choose healthier products to purchase. - Order healthier foods and beverages from a restaurant’s menu.
Topic 6 *Move Actively Anywhere!* • To increase physical activity by exercising according to the recommendations of the physical activity pyramid. • To do fun exercise.	Be active as much as possible, anywhere!	• Presentation: - Types of exercises to be performed indoors, especially at home or while sitting. - Advantages of a healthy lifestyle. - How to warm up before an exercise and cool down afterwards. • Activities: - Groups of students race to do physical activities fixed for every check point provided. - Aerobic exercise. - Wear pedometer during the activity and check the steps count upon finishing.
Topic 7 *Handle Perceptions Wisely, Accept the Uniqueness of Every Individual* • To identify ways to handle perceptions about one’s own body. • To accept the uniqueness and qualities of you and others.	Practice to achieve positive body image: i) Handle body perceptions wisely ii) Realize the uniqueness of others.	• Presentation: - Ways to handle perceptions and dissatisfaction with your own body. - Ways to prevent comparing between one’s own body and others’. • Activities: - List your physical characteristics and identify what others like about their bodies and qualities they are not satisfied with (if any). - Perform role-play to master ways to respond to statements of dissatisfaction by others. - Identify the qualities of a successful person. - Identify the similarities among your group members, as well their specific strengths and unique qualities. Share your findings with the other group members.
Topic 8 *Happy Practicing EPaL Lifestyle!* • To identify obstacles and solutions in relation to adopting a healthy lifestyle. • To enhance confidence in relation to adopting an EPaL-compliant lifestyle.	Happy practicing of EPaL lifestyle by eating healthy and balanced food, maintain a positive body image and an active lifestyle.	• Presentation: - Ways to gain confidence to maintain an EPaL lifestyle on the long run. • Activities: - Sharing the changes participants made over the course of this intervention. - Sharing the obstacles participants faced when practicing what they learnt in the EPaL programme. - In groups, participants recall one health message taken from EPaL and convey it to others. - Oath taking to adopt an EPaL-compliant lifestyle.

EPaL educational modules Version 1 and 2 focused on equipping peer educators with the necessary knowledge and skills to conduct sessions and educate others, whereas the activity book Version 1 and 2 was intended to serve as a guide for adolescents while performing the programme’s activities.

The Educational Materials for EPaL Version 1 and 2, as well as educational and activity cards and posters were developed and will be used by peer educators during interactive activities. The intervention consisted of two phases:

1. Phase 1: This phase comprised of topics 1–4 with the goal of providing adolescents with the basic knowledge and skills to adopt a healthy lifestyle.
2. Phase 2: This phase covered topics 5–8 with the goal of enabling adolescents to apply the knowledge and skills that they gained in Phase 1.

Peer educators received a Peer-Educator Training kits, which included EPaL modules, an activity book and related educational materials. The participants received an activity book and related educational materials.

A two-phase, 2-day training-of-trainer (TOT) course will be conducted to empower peer educators and provide them with knowledge and hands-on skills relevant to the intervention. TOT sessions were conducted by undergraduate and postgraduate students of nutrition and dietetics. Training for phase 1 will be carried out prior to the implementation of the first topic of the intervention. Training for phase 2 will be carried out after peer educators implemented the intervention activities of phase 1 at the school. The two phases are separated as such so that peer educators could better comprehend the content of the programme and deliver that content to their peers. Feedback from the facilitators will be given immediately after peer educators performed role playing to deliver EPaL topics as part of TOT. They will later asked to conduct eight intervention sessions before a small audience of their peers.

During these sessions at EPaL club, two peer educators will conduct the intervention in small groups of 5–7 participants. As a session begin, attendance will be taken, following which answer on the previous topic worksheet will be discussed. Then, the topic of the present session will be introduced and an ‘interaction session’ followed, in which presentations will be delivered by the peer educators and interactive activities will be demonstrated. Before a session concluded, the participants will be asked to set a goal to achieve on a personal level in tandem with the topic, and answer an exercise sheet designed to assess their understanding of the topic. Peer educators will conclude every session by handing out a feedback form to be filled by the participants to rate their satisfaction with the way the topic was presented. The participants also had to answer two questions in the form related to the topic of the session. The forms will be filled by the participants and collected by the peer educators.

### Measurements

All participants in both the intervention and the comparison schools will be assessed prior to the beginning of the programme (baseline assessment), after the final session of the intervention (post-intervention assessment) and 3 months afterwards (follow-up assessment).

#### Socio-demographic characteristics

Adolescents will answer questionnaires regarding their socio-demographic backgrounds, including gender, ethnicity, age, date of birth, number of family members living together, number of siblings, academic achievements and parents’ background (educational level, occupation and monthly income).

#### Anthropometric measurements

Anthropometric measurements will be carried out by trained personnel. Body weight will be measured using Tanita Glass Digital Bathroom Scale Model HD-382 (Tanita Corporation, Japan), while height will be measured by using SECA 206 height mechanical measuring tape (SECA, Germany). Body weight and height will be used for BMI calculation using the following formula:

$$ \mathrm{B}\mathrm{M}\mathrm{I} = \mathrm{weight}\ \left(\mathrm{kg}\right)/\ {\mathrm{height}}^2\left({\mathrm{m}}^2\right). $$

BMI-for-age (z-score) (BAZ) will be determined using BMI and height values. BAZ values will be used to categorize the status of each participant’s body weight in line with WHO Growth Reference for individuals 5–19 years old [30]. BAZ cut-off points are as followed: Severe thinness: <−3 SD; thinness: <−2 SD; normal weight: ≤ − 2 SD and ≥ 1 SD; overweight: > +1 SD; and obesity: > +2 SD.

Waist circumference will be measured by using a measuring tape. Abdominal obesity status will be determined by referring to the cut-off point of the 90th percentile of waist circumference of adolescents aged 12–16 years [31]. Omron HBF-306 Body Fat Analyzer Scale (Omron Corporation, Japan) will be used to measure the percentage of body fat. The instruments used were adequately calibrated; and adolescents were asked to remove their shoes and empty their pockets before measurement.

#### Stages of change

According to the Transtheoretical Model, five stages of change will be identified: Pre-contemplation, contemplation, preparation, action and maintenance for healthy diet [32], breakfast, food portion size, screen-viewing time (television/ video/ DVD) [33] and physical activity [34]. This model will be used to determine adolescents’ adherence to a healthy lifestyle before and after the implementation of the intervention. This will provided a clear view of the progress that the participants made in terms of adopting and maintaining health behaviours [35].

#### Disordered eating

The Eating Attitudes Test-26 (EAT-26) is a widely used instrument to assess eating disorders based on attitudes, feelings and behaviours [36] and will, hence, used in the present study. The EAT-26 consisted of 26 items with six response categories each, ranging from 1 (never) to 6 (always). A score higher than 20 indicated disordered eating. Items on EAT-26 scale allowed three subscales to be calculated, known as dieting; bulimia and food preoccupation and oral control.

#### Body image

Three instruments were used to assess dimensions of body image i.e., the perception of one’s body weight status [37], Body Dissatisfaction subscale and Body Importance subscale extracted from the Body Image and Body Change Inventory [38], and the Body Image Scale. The questionnaire used had been shown to be valid and reliable when used in studies that involve adolescents [38]. Perception of current weight status will be assessed by using parameters adapted and modified from Simko et al. [37]. Parameters will be ranked 1 (very thin) to 5 (obesity). Thereafter, adolescents’ perceptions of their current weight status will be compared to their actual weight status. Body image will be estimated based on the following calculation:

$$ \mathrm{Body}\ \mathrm{Image} = \mathrm{Perceived}\ \mathrm{body}\ \mathrm{size} - \mathrm{Actual}\ \mathrm{body}\ \mathrm{size} $$

A ‘correct-estimator’ will correspond to a score of 0; an ‘under-estimator’ will be identified when the score is < 0; and an ‘over-estimator’ referred to subjects with a score > 0.

Body Dissatisfaction subscale consisted of 10 items with five response categories, ranging from 1 (extremely satisfied) to 5 (extremely dissatisfied). Similarly, Body Importance subscale consisted of 10 items with five response categories, ranging from 1 (extremely important) to 5 (not important at all). Body Image Scale will also be used to assess adolescents’ satisfaction with their own body sizes. Nine body figures will be consulted, ranging from 1 (the thinness body size) to 9 (the biggest body size). Participants’ actual body size, perceived ideal body size and healthy body size will be identified. The discrepancy score will be calculated as follows:

$$ \mathrm{Satisfaction} = \mathrm{Perceived}\ \mathrm{current}\ \mathrm{body}\ \mathrm{size} - \mathrm{Perceived}\ \mathrm{ideal}\ \mathrm{body}\ \mathrm{size} $$

‘Satisfied with body size’ will correspond to a score of 0. ‘Dissatisfied with a desire to be larger’ will be implied by a score < 0, while ‘dissatisfied with a desire to be thinner’ will be implied by a score > 0.

#### Health-related quality of life (HRQoL)

Pediatric Quality of Life Inventory 4.0 (PedsQL™4.0) [39] will be used to assess health-related quality of life (HRQoL) among the adolescents using 23 items. Adolescents were asked to recall health-related problems experienced in the past month and rank the frequency, with 0 being never had a problem, and 4 being almost always had a problem. Scores of all items were plotted on a 0–100 point scale (0 = 100, 1 = 75, 2 = 50, 3 = 25, 4 = 0). Higher total PedsQL™ scores corresponds to better HRQoL.

PedsQL™ will be interpreted based on an ordered scale. The lower orders scale had four dimensions [40]: Physical Functioning (eight items), Emotional Functioning (five items), Social Functioning (five items) and School Functioning (five items). These four dimensions will be combined to derive a higher order scale comprising two dimensions: Physical Health (physical functioning subscale) and Psychosocial Health (emotional, social and school functioning subscales). PedsQL™ 4.0 had been shown to be valid and reliable to assess HRQoL in Malaysian adolescents [41].

#### Self-esteem

Rosenberg Self-esteem Scale (RSES) [42] will be used to measure adolescents’ self-esteem. The scale consisted of 10 items. Assessment assumes 1 as “strongly agree” and 4 as “strongly disagree”. RSES had been shown to be reliable and valid when used in studies involving adolescent groups [43, 44].

#### Pubertal stage

Pubertal Development Scale (PDS) [45] will be used to detect puberty in adolescents. Five characteristics will be assessed: spurt in height; pubic hair; skin changes; facial hair growth and voice change in males; and breast development and menarche in females. Except for menarche, to which a dichotomous response scale (‘yes’ or ‘no’) will be used, answers regarding puberty were provided by using a four-point Likert scale whereby 1 signifies no development, while 4 indicates that development was complete. Based on the given scores, the adolescents will be categorized into five pubertal groups: pre-pubertal, early pubertal, mid-pubertal, late pubertal and post-pubertal.

#### Eating behaviour

Two questionnaires will be used to assess adolescents’ eating behaviours: Three-Factor Eating Questionnaire-R18 (TFEQ-R18) [46, 47] and Eating Behaviour Questionnaire (EBQ) [48]. TFEQ-R18 will be used to determine current dietary practices. It measured three different aspects of eating behaviour: Cognitive restraint (tendency to constantly and consciously restrict food intake instead of using physiological cues, hunger and satiety as regulators of food intake), uncontrolled eating (tendency to overeat, with a feeling of losing control), and emotional eating (tendency to eat in response to negative emotions) [49]. Overall, TFEQ-R18 consisted of 18 items will be answered using a 4-point response scale whereby 1 is “definitely true” and 4 is “definitely false”.

EBQ will rather be used to assess main meal consumption and snacking behaviour, as well as family meals, eating outside and favouring take-away food. EBQ comprised of 11 items with 8 response categories ranging from 0 (0 day) to 7 (7 days). Adolescents will responded by stating the frequency of food intake in the past seven days.

#### Dietary intake

Food consumption, energy and nutrient intake will be assessed via 1-day dietary recall. Adolescents will be interviewed to determine the type and amount of foods and beverages they consumed on daily bases, as well as the food and beverage brands they preferred and the food preparation methods they used. Household measurements will be prepared to assist adolescents in estimating the quantity of food and beverages they consumed. The Nutritionist Pro™ Diet Analysis software will be used to assess energy, macronutrients and sugar intake. The software will also be used to estimate daily serving size of rice, bread, cereals, cereal products, vegetables, fruits, fish, poultry, meat, legumes, milk and dairy products.

#### Physical activity

Energy expenditure through physical activity will be assessed using one-day physical activity recall. Adolescents will be asked to recall all the activities they performed, including sitting, standing and walking, every 15 min during a 24-h period. Assessment of physical activity will be conducted on the same day of dietary recall. Every activity will be assigned an MET value based on the Compendium of Physical Activities (1 MET = 3.5 ml of oxygen/ kg body weight/ min or resting metabolic rate) [50, 51]. Energy expenditure during every activity will be calculated by multiplying the value of MET, the duration of the activity (hour) and body weight (kg) [50]. Total daily energy expenditure (TDEE) will be calculated by determining the sum of energy expenditure per activity per day. The physical activity level (PAL) of the participants will be calculated by dividing TDEE by the basal metabolic rate (BMR) [52], and will be classified into four categories: Sedentary (PAL < 1.40), light (PAL = 1.40–1.69), moderate (PAL = 1.70–1.99) and vigorous (PAL = 2.00–2.40) [52].

#### Knowledge, attitude and practice of healthy lifestyle

A Knowledge, Attitude and Practice of EPaL Lifestyle Questionnaire (KAP-ELQ) was developed prior to data collection to measure the construct of knowledge, attitude and practice in relation to healthy eating, physical activity and body image among adolescents. The instrument consisted of items adapted from standard questionnaires, literature reviews and text books. KAP-ELQ comprised 32 multiple-choice questions pertaining to knowledge. Additionally, 24 items with six response categories, ranging from 1 (strongly disagree) to 6 (strongly agree), will determine adolescents’ attitude towards a healthy lifestyle. Moreover, practice of a healthy lifestyle will be assessed by answering 35 items, 13 of which had five response categories whereby 1 meant “every day” and 5 meant “never”. The other 22 items also had five response categories; however, 1 implied “very often”, while 5 implied “never”. The items had been reviewed for suitability, relevance and accuracy by an expert panel comprising of nutritionists and health educators. Based on the feedback and recommendations by the expert panel, the items were either retained unchanged, revised or removed.

### Data analysis

Data will be analysed by using version 21 of the IBM SPSS Statistical software (IBM SPSS Statistics, Inc., Chicago, IL, USA). Depending on the distribution of the variable of interest, descriptive statistics of continuous data will be presented by using the mean and standard deviation, and the median and the inter-quartile range. Categorical data will be presented as frequencies and percentages. *T*-test will be used to detect differences between groups for continuous variables, while chi-square will be used to investigate possible correlations between categorical variables. The repeated measures analysis of variance (ANOVA) will be used to detect significant differences within the groups. The evaluation of the intervention will be based on an intention-to-treat analysis. Differences with *p*-values < 0.05 were considered significant.

## Discussion

The present work hypothesized that EPaL will effects positive change in body weight status and promotes healthy lifestyle. The quality of life in terms of health is expected to be significantly improves in peer educators and participants after the implementation of the programme. Adolescent peer leaders were found to be helpful and capable of educating young adolescents on aspects of nutrition, which resulted in positive behavioural changes in both the peer educators themselves and the participants [53]. Similar peer-led approaches were shown to be effective tools towards achieving better awareness, healthy behaviours and attitudes in children as young as 5 years old [22, 54] and promote self-efficacy in adolescents [55].

Peer leadership gives young people a sense of control and empowers them by promoting a feeling of social usefulness [56]. Adolescents testified that they preferred peer-led sessions to teacher-led ones as they were able to communicate well with peer leaders and appreciate the way peer leaders delivered the information [57].

Integrated interventions that tackle both obesity and eating disorder issues were shown to be effective previously [21, 22]. They were associated with improved physical activity, eating patterns, weight control behaviours, body image [33], knowledge, behaviour and attitude related to healthy living, and systolic blood pressure [22]. In the present work, when implementing an integrated intervention, EPaL, developed specially for Malaysian adolescents, it is expected to improve the health status of the adolescents, probably because EPaL was carefully developed to better suit a Malaysian setting based on field assessments and field data.

To the best of our knowledge, an intervention using a peer-led approach to prevent both overweight and disordered eating among adolescents had not been implemented in Malaysia. In many aspects, our intervention stands apart from existing obesity intervention programmes in Malaysia. It incorporated a body-image component to preventing overweight and disordered eating among adolescents. Inclusion of this component acknowledged that body dissatisfaction was a shared risk factor of both overweight and disordered eating [15]. The integrated approach of this intervention also had food intake and physical activity components and was found to be more time and cost-effective compared with separate programmes [19].

The present study has several strengths. It demonstrated the uniqueness of peer-led intervention approaches in promoting healthy lifestyle among Malaysian students. Advantages of the peer-led approach were highlighted in previous studies. Generally, peer educators testified that peer-led approach gave them a valuable opportunity for personal development and learning by build upon young people’s skills and abilities [56, 57]. It also resulted in greater improvement in healthy lifestyle practices [53].

This study also contributed educational modules, activity books and educational materials that could serve as references and teaching materials and which may be suitable for use by the Malaysian Ministry of Health, Malaysian Ministry of Education, NGOs, health professionals, schools and parents.

Yet, the present work has several limitations. For instance, randomisation was only performed when selecting the schools. The use of an experimental design that ensures randomisation while recruiting participants and assigning peer educators would be more favourable and may produce higher levels of confidence to term of the validity and causality of the programme’s effectiveness [58]. Nevertheless, the quasi-experimental design used in this study may have better mimicked a real-life situation. Quasi-experimental design allows researchers to study a phenomenon in a natural setting in real-time [59, 60]. Overall, the results of this study will add to the knowledge and evidence of the effectiveness of the health intervention. It may be useful as a model to develop future health and nutrition interventions for adolescents in Malaysia.

## Abbreviations

ANOVA: Analysis of variance
BMI: Body mass index
EAT-26: Eating Attitudes Test-26
EBQ: Eating Behaviour Questionnaire
EPaL: Eat Right, Be Positive About Your Body and Live Actively
HRQoL: Health-related quality of life
NEDC: National Eating Disorders Collaboration
PAQ-C: Physical activity questionnaire for older children
PedsQL™: Pediatric Quality of Life Inventory™
TFEQ-R18: Three-Factor Eating Questionnaire-R18
TOT: Training of trainers
